# Daily Consumption of an Anthocyanin-Rich Extract Made From New Zealand Blackcurrants for 5 Weeks Supports Exercise Recovery Through the Management of Oxidative Stress and Inflammation: A Randomized Placebo Controlled Pilot Study

**DOI:** 10.3389/fnut.2020.00016

**Published:** 2020-02-27

**Authors:** Roger D. Hurst, Kirsty A. Lyall, Robyn W. Wells, Gregory M. Sawyer, Dominic Lomiwes, Nayer Ngametua, Suzanne M. Hurst

**Affiliations:** ^1^Food Innovation Portfolio, The New Zealand Institute for Plant & Food Research Ltd., Palmerston North, New Zealand; ^2^Food Innovation Portfolio, The New Zealand Institute for Plant & Food Research Ltd., Hamilton, New Zealand

**Keywords:** blackcurrant anthocyanins, exercise, exercise recovery, oxidative stress, acute inflammation, IL-10

## Abstract

**Background:** Regular exercise is essential to a healthy lifestyle but evokes an oxidative and inflammatory stress. Depending upon its intensity and duration this can result in either beneficial adaptive changes or underlying tissue damage that impacts upon long-term health and individual sporting training schedules. Functional foods containing plant bioactives have potential to support exercise through management of the detrimental aspects of exercise and complement ergonomic adaptive benefits.

**Aim:** Previously we reported that a single consumption of a 3.2 mg/kg New Zealand blackcurrant anthocyanin-rich extract (BAE) 1 h before a 30 min rowing exercise attenuated moderate exercise-mediated oxidative stress and supported innate immunity. Here we evaluate whether the efficacy of a single consumption of BAE 1 h prior to exercise is changed after extended daily BAE consumption for 5 weeks.

**Results:** On week 1, a single consumption of BAE 1 h before a 30 min row mediated a significant (*p* < 0.05) 46% reduction in post-exercise-induced malondialdehyde (MDA) by 2 h compared to a 30% reduction in the placebo group. Similar efficacy was observed 5 weeks later after daily consumption of BAE. In addition, daily BAE consumption for 5 weeks improved the efficacy to (a) resolve acute inflammation, and (b) increased plasma IL-10, salivary beta-defensin 2 (BD2) and secretory IgA. Although no change in plasma antioxidant capacity was detected, a significant (*p* < 0.009) positive correlation between plasma IL-10 and plasma antioxidant capacity (*R*^2^ = 0.35) was observed on week 6 after 5 week BAE consumption suggesting IL-10 influences antioxidant properties. Using a differentiated myotubule cell-line revealed that whilst IL-10 had no direct antioxidant neutralizing action, longer-term exposure (24 h) attenuated 2,2′-Azobis(2-amidinopropane) dihydrochloride (AAPH)-induced myotubule oxidative stress, supporting a putative role for IL-10 in the modulation of cellular antioxidant systems.

**Conclusions:** Daily consumption of BAE for 5 weeks serves to enhance the exercise recovery effectiveness of a single consumption of BAE and promotes beneficial/protective antioxidant/anti-inflammatory cellular events that facilitate exercise recovery.

## Introduction

A diet rich in digestible plant polyphenols (i.e., fruits, vegetables, cereals) is vital to a healthy lifestyle and the prevention of poor lifestyle and age-related illnesses such as diabetes type-2 (1, 2). Polyphenolic compounds are usually characterized by their antioxidant properties and although the exact cellular action is still unclear, there is overwhelming evidence from a number of epidemiological, nutritional intervention and *in vitro* cellular studies, that the consumption of plant polyphenols supports long-term health through cell mechanisms that are unquestionably independent of their inherent chemical antioxidant properties. Anthocyanins are a major class of polyphenols that exhibit endogenous antioxidant properties important for plant health (3, 4), and have been shown to underlie the human health benefits of fruit/vegetable consumption through the activation of long-term adaptive processes (5, 6). In a feeding study reported by He and Giusti (7) the consumption of anthocyanins (160 mg of an anthocyanin extract, twice a day for 1–2 months) showed no adverse health effects, revealing that in most individuals anthocyanins are well-tolerated. Additionally, anthocyanin bioavailability is considered relatively low and although there is some evidence for tissue accumulation, it is still debatable whether dietary anthocyanins *per se* or their downstream metabolites are actually responsible for their beneficial health properties (4, 8–10). A common health outcome from the long-term consumption of anthocyanin-rich foods is the regulation of cellular oxidative stress/inflammation, which usually involves the up-regulation and/or maintenance of adaptive health-promoting processes within the body, rather than being mediated by the chemical antioxidant properties of the digested anthocyanins. Human epidemiological studies (6) reveal that daily consumption of foods rich in anthocyanins supports the maintenance of cellular antioxidant capacity and alleviates oxidative stress in a number of chronic diseases (11–13). Several feeding studies report an increase in plasma antioxidant capacity after the consumption of berryfruit rich in anthocyanins, e.g., blackcurrants (14, 15), strawberries (16), blueberries (17), and blackberries (18). Similarly, nutritional intervention studies by others (19–21) reveal that the consumption of blackcurrant anthocyanins exhibit cardiac and eye health as well as alleviating oxidative stress in both physiological and disease scenarios. Anthocyanins have been shown to reduce cellular oxidative damage and inflammation through a number of cellular mechanisms, including the upregulation of cellular c*yclic adenosine monophosphate* responsive element driven upregulation of reduced g*lutathione* content (22), nuclear redox factor/antioxidant responsive element (nrf2/ARE)-induced antioxidant enzyme expression, e.g., superoxide dismutase, catalase (23, 24), and down-regulation of nuclear factor kappa-light-chain-enhancer of activated B cells (NF-κB) and activator protein-1 (AP-1)-induced inflammation (25–27). Therefore, the improved lifestyle health benefits from consuming foods rich in anthocyanins appear to involve the activation of different cellular pathways that contribute to a dynamic cellular antioxidant/anti-inflammatory microenvironment capable of responding to redox fluctuation caused by various physiological (e.g., exercise) or pathophysiological (e.g., atherosclerosis) events.

Regular exercise is an essential element of a healthy lifestyle, where at least 30 min of daily moderate exercise is considered enough to maintain long-term health and well-being (28, 29). Physical exercise, however, evokes an acute oxidative and inflammatory response that, depending upon its intensity and duration, can result in hormetic events (30) that result in either beneficial adaptive changes or sub-clinical tissue damage and delayed recovery. This may have impact upon an individual's health and further their ability to engage in strenuous exercise, which is an important training consideration for both competitive and non-competitive athletes (31). The inclusion of “functional foods,” drinks and supplements rich in potential antioxidants (e.g., polyphenols) has therefore been experimentally incorporated into sports training strategies with mixed success (32–34). A major consideration for the inclusion of a potential exercise-targeted functional food is that not only must it manage the detrimental aspects of exercise but ideally it should also complement the health and ergonomic adaptive benefits of exercise. Moreover, physical exercise causes a physiological shift in cellular redox status through an increase in reactive oxygen species (ROS)/reactive nitrogen species which is vital for the activation of cell signaling that supports the beneficial adaptive health and ergonomic outcomes. Preventing exercise-induced changes in cell redox status is likely to inhibit/delay adaptation to exercise (31, 35). Several studies have explored the beneficial efficacy of feeding anthocyanin-rich foods/supplements at various times before the exercise, and by upregulating cellular adaptive antioxidant systems rather than a direct suppression of oxidative stress (33, 36). For example, the consumption of pomegranate juice daily for 7 days before a 30 min treadmill (50% W_max_) exercise was shown to alleviate exercise-induced oxidative stress by upregulating adaptive antioxidant systems (37), and Morillas-Ruiz et al. (38) reported that supplementing cyclists with a berryfruit concentrate rich in polyphenols 15 min before a 90 min cycle (70% VO_2max_) similarly reduced exercise-induced oxidative stress via an adaptive antioxidant process. Therefore, since selected polyphenols, and maybe especially anthocyanins, may activate the same adaptive cellular signaling pathways as physical exercise itself, it is feasible that the consumption of these putative functional foods, at an appropriate time in relation to exercise, may actually complement these adaptive events and the benefits of exercise, and support exercise performance.

Incorporating and using anthocyanin-rich foods/supplements with efficacy in supporting the benefits of exercise and training is still in its infancy. Various factors of variable importance, including the type, intensity and duration of the exercise, the anthocyanin type/mix, its source, dose, and bioavailability/bioactivity will affect the desired outcome e.g., physiological adaptation, recovery from exercise-induced oxidative stress and/or muscle damage, and/or enhanced sporting performance. Nutritional timing i.e., a single dose consumption timed before or after exercise dependent upon the dynamics of the bioactives appearing in the blood, or repeated daily, weekly (time of day) consumption for a defined required period, will also likely affect the desired outcome. In the context of blackcurrant anthocyanins for exercise benefits exercise intervention studies (20, 39, 40) have demonstrated that pre-consumption for a variable set period before exercise facilitates exercise recovery but also has the potential to support other physiological events (i.e., cardiac health). These studies have utilized different pre-exercise consumption periods from a single consumption 1 h before exercise to 7 days of daily consumption before exercise. In a previous study by us, we found that following the single consumption of a New Zealand blackcurrant anthocyanin-rich extract (BAE, containing >0.8 mg/kg total anthocyanins) that the anthocyanins were first detectable in plasma 1 h following consumption. Utilizing these findings we found in a subsequent exercise intervention trial that consumption of BAE (containing >1.6 mg/kg total anthocyanins) 1 h prior to a 30 min moderate exercise indeed attenuated oxidative stress and supported innate immunity (41).

In this current study, we extend our latest findings and evaluate whether the efficacy of a single dose of BAE consumed 1 h prior to exercise (30 min row at 70% VO_2max_) endures, is enhanced, or becomes lost following daily BAE consumption for an extended period of time−5 weeks. We hypothesize that daily consumption of BAE for 5 weeks will modulate cellular adaptive processes that serve to either maintain and/or enhance the exercise recovery effectiveness of consuming BAE 1 h prior to exercise.

## Materials and Methods

### Human Trial Design

This study was carried out within The New Zealand Institute for Plant & Food Research Limited (Plant & Food Research) and was approved by the Northern Ethical Regional Committee, Hamilton, New Zealand (NYT/09/105). All subjects gave written informed consent in accordance with the Declaration of Helsinki.

#### Participant Selection

Healthy individuals between 20 and 60 years old were recruited from the Hamilton, Waikato community, and provided written informed consent. All recruited individuals agreed to (i) keep a food diary over the 6 week period of the study, (ii) refrain from taking dietary supplements rich in antioxidants over the period of the study and (iii) on weeks 1 and 6 of the study, agreed to refrain from consuming foods (including drinks) that were rich in both anthocyanins and antioxidants (list of foods provided by trial coordinator) 24 h before a timed nutrition-exercise trial. In addition, participants agreed to complete a fitness assessment, which included the completion of a questionnaire described by Baecke et al. (42) (i.e., assessment of individuals' habitual physical activity) and taking part in a pre-study rowing exercise trial, which not only familiarized them with the rowing technique required but also enabled the trial coordinator to determine the rowing intensity required by the participants to achieve 70% VO_2max_. Participants were excluded from the study if they had known fruit allergies, blood-borne diseases (e.g., hepatitis), viral or bacterial illness, were undergoing immunization, taking medication that involved antioxidant rich medicines, had defective blood properties (e.g., clotting), and/or were pregnant or planning to become pregnant. Individuals participating in the exercise trial were also excluded if they were unable to perform the 30 min rowing exercise (e.g., current injury or recovering from injury received within the last 3 months). Participants selected for this study showed similar moderate daily physical activity scores on the Baecke questionnaire as described in our previous BAE study (41).

#### Nutritional Intervention

The blackcurrant anthocyanin extract (BAE) consisted of 34% anthocyanins and can be sourced from the New Zealand Blackcurrant Co-operative (www.nzblackcurrants.com/, New Zealand). The extract was made from New Zealand blackcurrants and contained four dominant anthocyanin glycosides (90%); delphinidin-3-O-glucoside, delphinidin-3-O-rutinoside, cyanidin-3-O-glucoside and cyanidin-3-O-rutinoside (39). The BAE extract displayed a high inherent antioxidant capacity (oxygen radical absorbance capacity [ORAC]; 6,424, ferric reducing ability of plasma [FRAP]; 3,810 μM Trolox equivalents) compared to other berryfruit (43), which is due to its high anthocyanin content as the extract contained <1 mg/100 g of vitamin C. In this current study, we selected to use a dose of BAE (equ. 240 mg total anthocyanins or 3.2 mg/kg) that was greater than the reported minimal effective dose (41) and has been previously repeatedly shown to facilitate recovery from exercise-induced oxidative stress (39, 41). The dose of extract used aligns well with a typical dietary serving and equates to ~48 g of blackcurrant fruit and 14 mL of blackcurrant juice concentrate (65Brix). To obtain the required total anthocyanin dose of 3.2 mg/kg (~240 mg) for each participant in this current study, the amount of blackcurrant extract given was calculated using the participants' body weight and the total amount of anthocyanins (mg) in the blackcurrant extract. The total anthocyanin content of the BAE batch used in this study was calculated from the HPLC analysis as previously described by Lyall et al. (39). A sugar (glucose) placebo (PLA) which was also encapsulated and matched to the sugar content of the extract was used. This PLA was selected to exclude any possible acute increases in plasma antioxidant capability being due to sugar metabolism (44) and has been used in previous nutrition exercise intervention trials by us and others (39, 41, 45).

#### Trial Format

**Figure 1:**
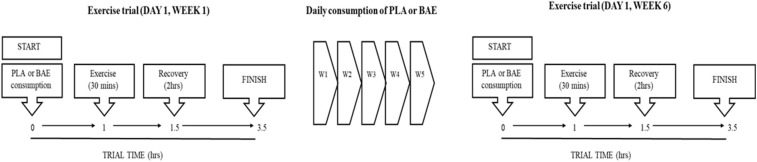
Flow chart of the 6 week study design to examine the efficacy of daily blackcurrant anthocyanin-rich extract (BAE) consumption for 5 weeks on the recovery from exercise-induced oxidative stress and systemic immune defense biomarkers. PLA, placebo.

The study design is based upon a previous trial examining the time- and dose-dependent efficacy of pre BAE consumption on exercise recovery (41). A double-blind placebo-controlled trial design consisted of two groups: PLA or BAE extract consuming the equivalent of 3.2 mg/kg total anthocyanins. The study was repeated in two separate trials containing 18 participants per trial (36 participants in total) which were matched according to age and weight. Averages ages were 40.6 and 36.7 year (range 25–58 year for both trials) and weights 73.9 and 75.3 kg (range 59–94 kg for both trials) for trials 1 and 2, respectively. Participants were randomly assigned to either the PLA or BAE study arms (18 participants per treatment arm in total), by an independent Plant & Food Research employee using a random-number generating Microsoft software computer programme, and blinded from both trial participants and the trial coordinator. The study (Figure 1), involved blinded participants consuming 2 opaque vegetable capsules (with 100 mL water) that contained either BAE (3.2 mg/kg total anthocyanins) or PLA and then 1 h later performing a 30 min rowing exercise (using a Concept 2 rower) at 70% VO_2max_, previously described by our group (41, 46). Blood and saliva samples were taken at set periods before and after this timed nutrition-exercise trial. Following this participants were asked to consume two capsules (PLA or BAE) every morning for the next 5 weeks. At the beginning of week 6, participants were asked to repeat the timed nutrition-exercise trial they performed on week 1. To maintain continuity, participants performed the trials on week 1 and 6 at the same time of day (~0800 h) and all participants consumed a set breakfast (consisting of a One-Square Meal^TM^ cereal bar and drink) 2 h before the start of the trial.

#### Sampling

Blood and saliva samples were collected at set time periods on weeks 1 and 6 of the study. Venous blood was collected into EDTA-coated tubes from the participants 1 h after the consumption of the PLA or BAE (i.e., pre-exercise), immediately after completing the 30 min row and then again after the 2 h recovery. The collected blood was immediately centrifuged (300 g, 18°C, 10 min) and the plasma samples were then aliquoted (8 × 0.5 mL), snap frozen in liquid nitrogen and then stored at −80°C until assessed for oxidative stress, antioxidant, inflammatory indices and the assay's co-efficient of variation. Saliva was collected before the consumption of either BAE or PLA. Participants were asked to rinse their mouth with water and then spit into a plastic tube. The saliva was centrifuged (300 g, 18°C, 10 min), and the soluble phase was aliquoted, snap frozen in liquid nitrogen and frozen at −80°C until measurement of inflammatory indices.

### Biochemical Measures

#### Oxidative Stress Parameters

Plasma oxidative stress status was assessed by the concentrations of the lipid oxidation product malondialdehyde (MDA) and plasma oxidative-generating capability: ROS and superoxide (SO).

- **Lipid peroxidation concentrations** were assessed using either a commercial MDA ELISA kit (plasma, Cayman Chemicals, Redfern, Australia) or by HPLC (myotubule experiments) described by Lomiwes et al. (47). Plasma MDA concentrations were calculated, against MDA standards (0.625–50 μM) and presented as μM plasma MDA. The average % coefficient of variance for these assays was 16%.
- **Plasma ROS-generating capability** was assessed using a hydrolysed carboxy-dihydro-2′, 7′-dichlorohydrofluorescein diacetate fluorophore (carboxy-H_2_DCFDA, Molecular Probes, Life Technologies, Auckland, New Zealand) in a kinetic assay previously described (39, 41). Briefly, hydrolysed carboxy-H_2_DCFDA and a threshold (1 μM) H_2_O_2_ concentration, determined prior to assessing trial plasma samples, was added to diluted plasma in phosphate buffered saline, pH 7.4 (1:4 dilution). The change in fluorescence intensity (FI) was measured over 5 min at RT using a fluorescence plate reader (BMG FluoStar Optima; Alphatech Systems, Auckland New Zealand) with excitation and emission wavelengths set at 485 and 520 nm, respectively. Data were calculated as the difference in FI (FI_5min_–FI_0min_) minus plasma/ PBS control (ΔFI_5min_) and results expressed as ΔFI_5min_. The average % coefficient of variance for this assay was 9%.
- **Plasma superoxide-generating capability** was assessed using a modified nitroblue tetrazolium assay described by Esfandiari et al. (48), which is based on the conversion of water-soluble yellow-colored dye to its blue formazan product by superoxide anions. Briefly, plasma (diluted 1:4 in a phosphate buffer, pH 5.6) was added to the nitroblue tetrazolium substrate in the presence of β-nicotinamide adenine dinucleotide, and the conversion to the formazan product was assessed by measuring the change in absorbance 560 nm over 15 min in a BMG FluoStar Optima fluorescence plate reader at RT in the presence of a threshold concentration (4 μM) of phenazine methosulfate (PMS); determined prior to the assessment of trial plasma samples. Data were calculated as the change of absorbance over 10 min minus the plasma/buffer control and results expressed as ΔAbs_560nm_. The average % coefficient of variance for this assay was 7%.

#### Antioxidant Measures

The ferric-reducing ability of plasma (FRAP) was measured using the standard method described by Benzie and Strain (49). Briefly, plasma diluted in acetate buffer (1:5 dilution) was added to an equal amount of FRAP reagent (containing TPTZ [2,4,6-tripyridyl-s-triazine, Sigma-Aldrich] and ferric chloride in a hydrochloric acid solution). After 15 min of incubation at RT, the absorbance was measured in a fluorescence plate reader (BMG FluoStar Optima) set at wavelength 593 nm. Plasma antioxidant capacity was measured against a standard curve of Trolox (Merck NZ), calculated as μM Trolox equivalents and data presented as mg/mL Trolox equ. The average % coefficient of variance for this assay was 3%.

#### Inflammatory Mediators

Plasma and salivary innate immune mediators were measured using specific sandwich ELISA kits (purchased from either R&D systems, Invitro Technologies, Auckland, New Zealand or BioLegend, MediRay, Auckland, NZ). Using ELISA standards, data were calculated and presented as appropriate weight/volume, e.g., pg/mL. The average % coefficient of variance for the various inflammatory mediator assays was 6%, with 4% being the lowest and 10% being the highest.

### Skeletal Muscle Myotubule Cell Oxidative Stress Models

Mouse C2C12 skeletal muscle myoblasts (purchased from the European Collection of Animal Cell Cultures, Sigma-Aldrich, Australia) were grown in complete DMEM media containing 20% heat-inactivated fetal calf serum (FCS). Differentiation of myoblasts into myotubules was performed by incubating cells in DMEM media containing 2% FCS for 6 days. The myotubule population was then used to assess the antioxidant properties of interleukin-10 (IL-10).

#### ROS Generation

To assess the direct ROS-neutralizing capability of IL-10, myotubules were first incubated with 100 μM carboxy-H_2_DCFDA diluted in DMEM media containing 2% FCS for 30 min. The cells were then washed twice in media and then simultaneously stimulated with 5 mM 2,2′-Azobis 2-amidinopropane dihydrochloride (AAPH, Sigma-Aldrich) in the absence or presence of N-acetylcysteine (0.1–10 μM) or IL-10 (0.1 or 10 ng/mL). The change in fluorescence intensity (FI) was measured over 30 min at RT using a fluorescence plate reader (BMG FluoStar Optima) with excitation and emission wavelengths set at 485 and 520 nm, respectively. In cell experiments examining the indirect effect of IL-10 on ROS generation, myotubules were pre-incubated in the absence or presence of IL-10 (0.1 or 10 ng/mL) for 24 h. The cells were then washed, loaded with carboxy-H_2_DCFDA and the generation of ROS was evoked with 5 mM AAPH and monitored as described above. Data were calculated as the change in FI over 30 min or calculated as a change in FI after 10 min (FI_10min_–FI_0min_) and presented as % AAPH control.

#### Oxidative Stress Indices

Initial experiments were performed to optimize AAPH-induced protein carbonyl (protein oxidation) and lipid peroxidation (MDA) concentrations in the myotubule cell model described above. A 60 min incubation (37°C) of myotubules with 25 mM AAPH evoked an increase in cellular carbonyls (Sigma-Aldrich, Castle Hill, Australia) and MDA (Abcam, Melbourne, Australia) concentrations that was measureable using commercial kits. The direct effect of IL-10 was assessed by simultaneously incubating myotubules in the absence or presence of 0.1 or 10 ng/mL IL-10 and 25 mM AAPH for 1 h, whereas the indirect action of IL-10 was examined by pre-incubating myotubules in the absence or presence of IL-10 (0.1 or 10 ng/mL) for 24 h before AAPH (25 mM) stimulation. Baseline cellular oxidative stress indices, protein carbonyls and MDA were also examined after an hour of incubation in the absence of AAPH. In each case, the cells were then washed with PBS and processed for measuring cellular protein carbonyl and MDA concentrations following manufactures' instructions. Results were calculated as mmol/mg protein or nM for total cellular carbonyls or MDA levels, respectively.

### Statistical Analysis

Results are shown as means ± standard error or standard errors of the means (SEM) for the indices measured within each treatment group, and at least five separate experiments exploring the antioxidant properties in a mouse myotubule cell model. Statistical significance for the comparison between two groups was assessed using a paired Student's *t*-test. Multiple comparisons were assessed by ANOVA; one-way or two-way, using Microsoft Excel Analysis ToolPak statistical analysis software. Data showing significance in the ANOVA tests were further analyzed using either Scheffe or Tukey *post-hoc* analysis set at *p* < 0.05. In addition, the relationship between plasma antioxidant capacity and immune mediators was examined by regression analysis on two sets of variables. A *p* < 0.05 with a confidence level of 95% was regarded as statistically significant.

## Results

### Subject Analysis

Nutritional compliance by trial participants was maintained through mobile phone text and by meeting the trial coordinator at Plant & Food Research once a week to receive their weekly nutritional interventions (i.e., opaque vegetable capsules containing either 3.2 mg/kg BAE or equivalent sugar placebo: Average participant weight = 75 ± 9 kg. Over the 5 weeks all participants complied with the study's criteria and no adverse effects were reported. In addition, analysis of participants' food diaries showed that all subjects had maintained their normal diet and no obvious group differences were observed in terms of the consumption of foods and drinks rich in polyphenolic compounds and antioxidants. All participants omitted dietary supplements high in antioxidants from their diet over the 5 weeks of the study and complied with dietary requirements prior to each exercise. The menstrual cycle of women participants were not controlled over the period of the study. Of the original 36 participants recruited for this study, two withdrew from the first trial because of employment relocation and due to travel distances were unable to continue. Thirty-four healthy individuals (13 females, 21 males) aged between 25 and 58 years, with a mean age of 38 ± 11 years, completed the study, and were able to maintain their usual diet and exercise regime over the 5 weeks and reported no injury or illness. In addition, before the start of the study, all participants took part in an initial familiarization session [applying the protocol outlined in Lyall et al. (39) and Hurst et al. (46)] to optimize the rowing intensity required to evoke an oxidative stress after 30 min (indicated by post-exercise increases in plasma MDA and oxidative generating potential). All subjects recruited in this study showed an increase in these oxidative stress indices after a 30 min row and subjectively reported no adverse health effects or muscle pain or tenderness the following day.

### Recovery From Exercise-Induced Oxidative Stress

#### Plasma MDA Levels (Figure 2)

The 30 min exercise evoked ~2-fold significant (*P* < 0.01) increase in plasma MDA levels in both treatment groups. In week 1, consumption of BAE 1 h prior to exercise significantly (*p* < 0.05) reduced plasma MDA observed after 2 h recovery compared to the PLA group (1.38 ± 0.23 vs. 0.94 ± 0.13 μM, PLA vs. BAE) Repeat of the exercise session on week 6, after daily consumption of BAE for 5 weeks, revealed that consuming BAE 1 h prior to exercise showed the same efficacy in reducing post-exercise plasma MDA levels after 2 h recovery (0.76 ± 0.09 μM), which was significant from PLA (1.48 ± 0.21 μM), and not significantly different from week 1 levels.

**Figure 2:**
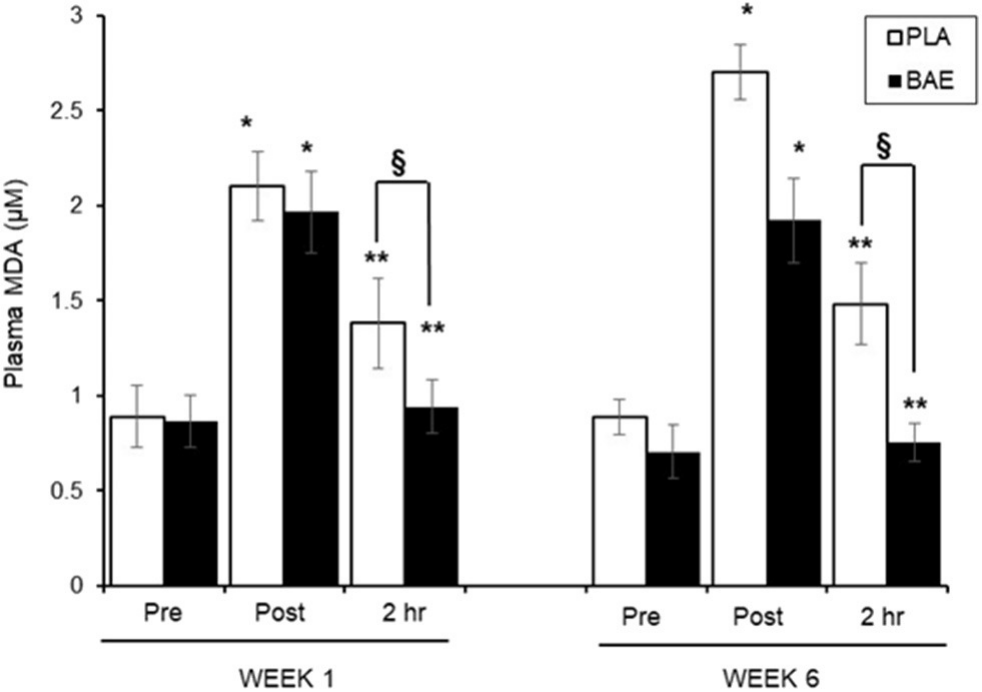
The influence of blackcurrant anthocyanin-rich extract (BAE) or placebo (PLA) consumption 1 h prior to exercise on plasma malondialdehyde (MDA) levels on week 1 and 6 after 5 weeks daily consumption. Results are expressed as mean ± SEM, *n* = 18 individuals per group. *p < 0.05 represents statistical difference from corresponding pre-exercise values. **p < 0.05 represents statistical difference from corresponding post-exercise values. ^§^*p* < 0.05 represents statistical difference between PLA and BAE values.

#### Plasma ROS-Generating Capability (Figure 3A)

In week 1, consumption of BAE 1 h prior to exercise caused a significant (*p* < 0.05) reduction in exercise-induced plasma ROS-generating capability after 2 h recovery compared to PLA (2,454 ± 5.75 vs. 1,511 ± 178 ΔFI_5min_, PLA vs. BAE). Repeat of the exercise trial on week 6, after 5 weeks of daily consumption of BAE showed that the pre-consumption of BAE 1 h prior to exercise retained its ability to reduce exercise-induced plasma ROS-generating capability after 2 h recovery compared to PLA (2,018 ± 262 vs. 1,185 ± 165 ΔFI_5min_, PLA vs. BAE). Moreover, no significant difference (*p* > 0.05) was observed in BAE efficacy between week 1 and 6 at this recovery time point.

**Figure 3:**
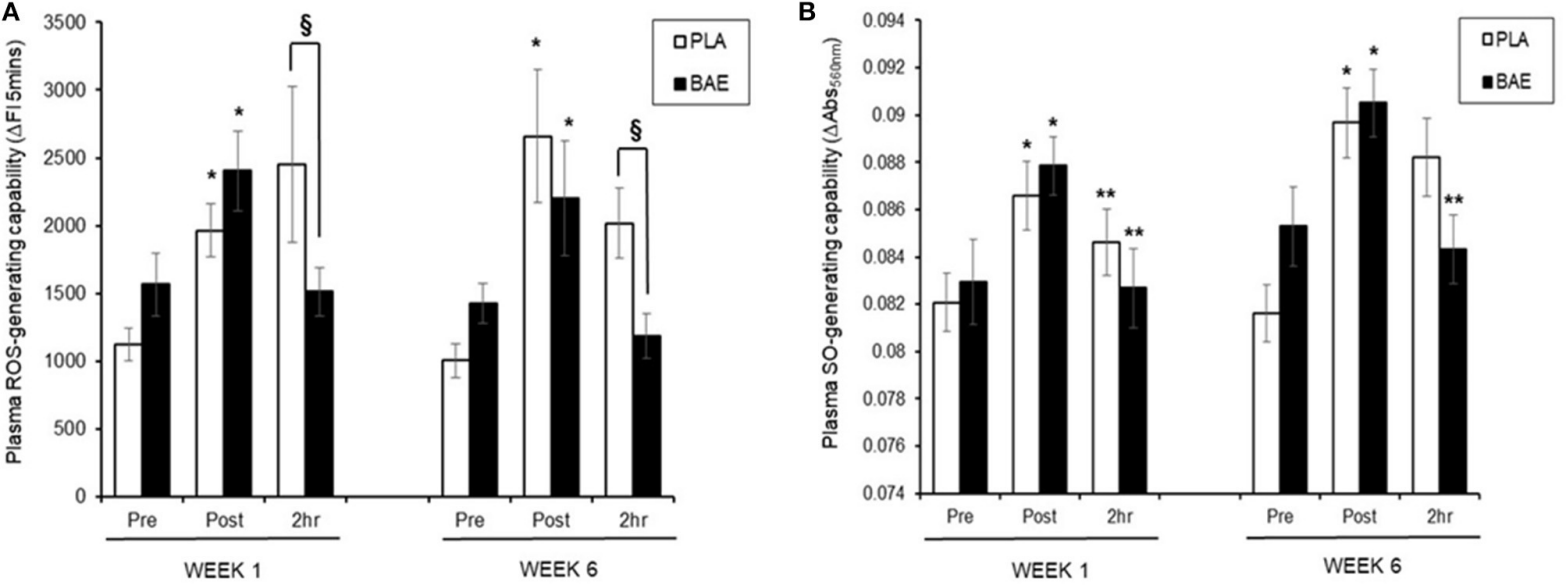
The impact of blackcurrant anthocyanin-rich extract (BAE) or placebo (PLA) consumption 1 h prior to exercise on plasma **(A)** reactive oxygen species (ROS)- and **(B)** superoxide anion (SO)-generating capability on week 1 and 6 after 5 weeks of daily consumption. Results are expressed as mean ± SEM, *n* = 18 individuals per group. *p < 0.05 represents statistical significance from corresponding pre-exercise values. **p < 0.05 represents statistical difference from corresponding post-exercise values. ^§^*p* < 0.05 represents statistical difference from PLA and BAE values.

#### Plasma SO-Generating Capability (Figure 3B)

In week 1, consumption of either PLA or BAE 1 h prior to the 30 min row caused a significant (*p* < 0.05) ~1-fold increase in plasma SO-generating capability, which was significantly (*p* < 0.05) reduced after 2 h recovery: PLA (0.87 ± 0.001 vs. 0.084 ± 0.001 ΔAbs_550nm_, post-exercise vs. 2 h recovery), BAE (0.088 ± 0.002 vs. 0.082 ± 0.002 ΔAbs_550nm_, post-exercise vs. 2 h recovery). Daily consumption of PLA or BAE 5 weeks showed exercise-induced plasma SO-generating capability and post-exercise recovery profiles as observed in week 1.

### Resolution From Exercise-Induced Acute Inflammation

#### Plasma TNFα (Figure 4A)

The 30 min row evoked a significant (*p* < 0.05) transient ~3-fold increase in plasma TNFα. In week 1, consumption of BAE 1 h prior to exercise significantly (*p* < 0.05) reduced the level of plasma TNFα measured after 2 h recovery compared to PLA (9.32 ± 1.39 vs. 3.69 ± 1.09 pg/mL, PLA vs. BAE). Repeat of this exercise on week 6, after daily BAE consumption for 5 weeks, also showed a significant (*p* < 0.05) decrease in post-exercise plasma TNFα levels in participants who had consumed BAE 1 h prior to exercise (7.41 ± 1.36 vs. 1.20 ± 0.8 pg/mL, PLA vs. BAE). Furthermore, the reduction in 2 h post-exercise plasma TNFα levels observed in week 6 was significantly (*p* < 0.05) lower than that measured in week 1.

**Figure 4:**
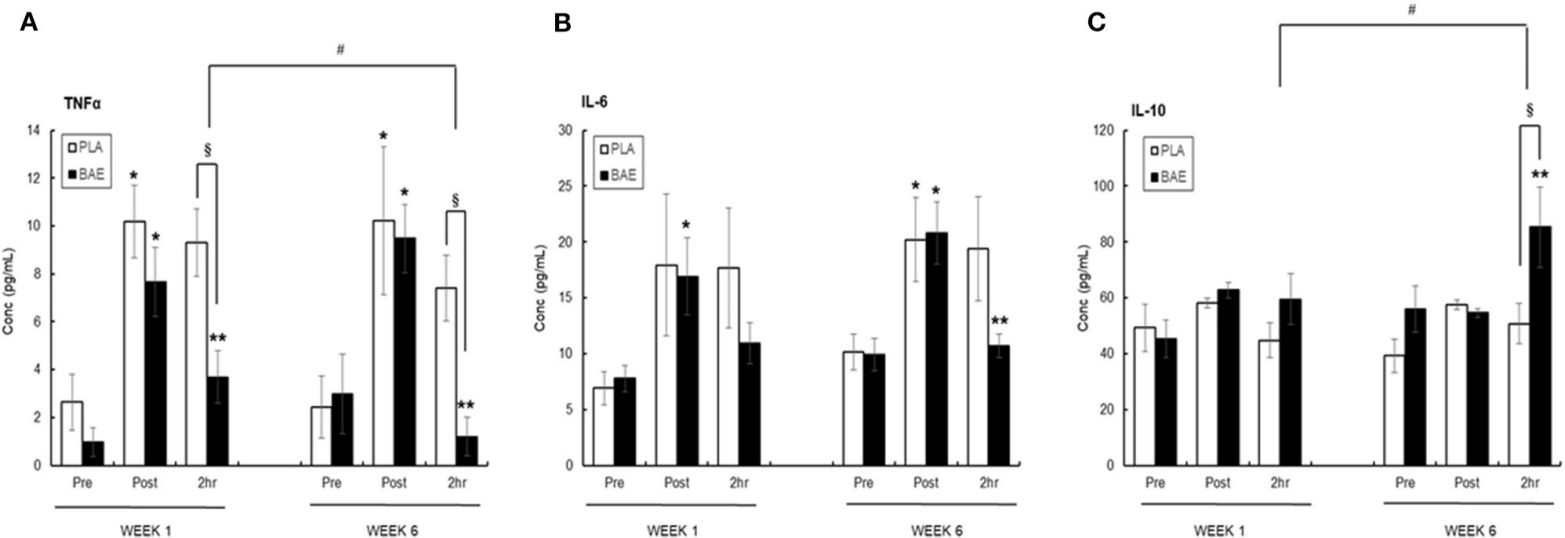
The effect of blackcurrant anthocyanin-rich extract (BAE) or placebo (PLA) consumption 1 h prior to exercise on plasma **(A)** TNFα, **(B)** IL-6, and **(C)** IL-10 on week 1 and 6 after 5 weeks of daily consumption. Results are mean ± SEM, *n* = 18 individuals per nutritional group. *p < 0.05 represents statistical significance from corresponding pre-exercise values. **p < 0.05 represents statistical difference from corresponding post-exercise values. ^§^*p* < 0.05 represents statistical difference between PLA and BAE. ^#^*p* < 0.05 represents statistical difference between corresponding week 1 and 6 values.

#### Plasma IL-6 (Figure 4B)

Completion of the 30 min row caused a ~2-fold increase in plasma IL-6, which in the PLA group was still elevated after 2 h recovery. In week 1 consumption of BAE 1 h prior to exercise caused a reduction in plasma IL-6 levels detected after 2 h recovery (17.68 ± 3.36 vs. 10.93 ± 1.87 pg/mL, PLA vs. BAE). A similar 2 h post-exercise effect was observed after daily consumption of BAE for 5 weeks (19.39 ± 4.65 vs. 10.68 ± 1.06, PLA vs. BAE). However, no significant difference (*p* > 0.05) from PLA was observed in post-exercise plasma IL-6 levels measured on either weeks 1 or 6.

#### Plasma IL-10 (Figure 4C)

The 30 min row had no effect on plasma IL-10 levels immediately after completion of the exercise or after 2 h recovery. In week 1, consumption of BAE 1 h prior to exercise had no effect on 2 h post-exercise plasma IL-10 levels (44.82 ± 6.12 vs. 59.50 ± 9.08 pg/mL, PLA vs. BAE). In contrast, daily consumption of BAE for 5 weeks prior to a repeat of the nutrition intervention exercise session on week 6 revealed a significant (*p* < 0.01) increase in post-exercise plasma IL-10 measured after 2 h recovery compared to PLA (50.72 ± 7.24 vs. 85.27 ± 14.42 pg/ml, PLA vs. BAE). Furthermore, the increase in plasma IL-10 observed after 2 h recovery in week 6 was significantly (*p* < 0.05) higher than that measured in week 1.

### Changes in Innate Immune Defense Mediators

**Figure 5:**
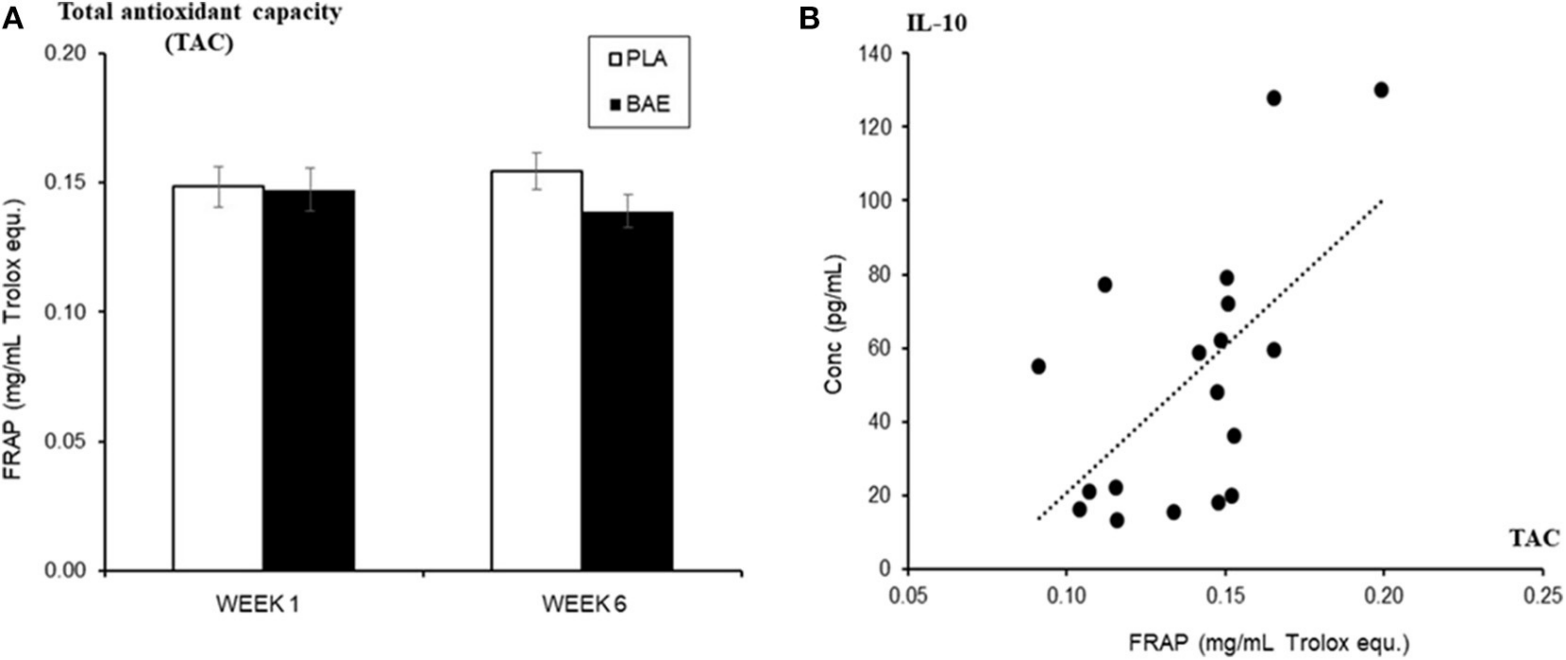
The influence of blackcurrant anthocyanin-rich extract (BAE) or placebo (PLA) consumption 1 h prior to exercise on **(A)** plasma antioxidant capacity (ferric reducing ability of plasma [FRAP]) on week 1 and 6 after 5 weeks of daily consumption, **(B)** its relationship (regression scatter plot) to plasma IL-10 levels on week 6. Results are mean ± SEM, *n* = 18 individuals per nutritional group. *R*^2^ = 0.35, *p* < 0.009.

Daily consumption of BAE altered the levels of some plasma and saliva immune biomarkers over 5 weeks (Table 1). A significant (*P* < 0.05) increase in immune modulatory plasma IL-10 and salivary mucosal anti-bacterial defense proteins (BD2 and secretory IgA) were observed in week 6 compared to week 1. Since IL-10 is associated with antioxidant status (50), we examined the relationship between plasma IL-10 and plasma antioxidant capacity. As shown in Figure 5A, the daily consumption of BAE for 5 weeks had no significant effect (*p* = 0.19) on plasma FRAP antioxidant capacity (0.154 ± 0.007 vs. 0.138 ± 0.006 mg/mL Trolox equ., PLA vs. BAE). However, comparison between FRAP antioxidant capacity and plasma IL-10 measured in the BAE group at week 6 (Figure 5B) revealed a significant positive correlation (*R*^2^ = 0.35, *P* = 0.009).

**Table 1:** Inflammatory mediators were measured on week 1 and 6 after daily consumption of either New Zealand blackcurrant anthocyanin-rich extract (BAE) or placebo (PLA) for 5 weeks.

**Nutritional intervention**		**Time**	
		**Week 1**	**Week 6**
Plasma C-reactive protein	PLA	0.96 ± 0.20	1.70 ± 0.75
(mg/L)	BAE	2.04 ± 0.75	2.10 ± 0.64
Plasma IL-6	PLA	6.91 ± 0.48	10.14 ± 1.59
(pg/mL)	BAE	7.78 ± 1.22	9.94 ± 1.49
Plasma IL-10	PLA	49.32 ± 8.48	41.74 ± 5.99
(pg/mL)	BAE	40.02 ± 6.49	53.67 ± 8.58*
Plasma IL-8	PLA	2.16 ± 1.08	4.98 ± 1.52
(pg/mL)	BAE	2.12 ± 0.58	2.54 ± 0.82
Plasma IL-17A	PLA	0.46 ± 0.06	0.49 ± 0.05
(pg/mL)	BAE	0.66 ± 0.17	0.82 ± 0.19
Plasma IL-27	PLA	0.67 ± 0.21	0.70 ± 0.19
(ng/mL)	BAE	0.70 ± 0.17	0.82 ± 0.19
Plasma TGFβ	PLA	6.99 ± 0.64	8.99 ± 1.17
(ng/mL)	BAE	10.11 ± 1.09	9.26 ± 0.68
Salivary BD2	PLA	990.04 ± 91.28	1,114.35 ± 96.1
(pg/mL)	BAE	928.69 ± 78.47	1,330.15 ± 135.18*
Salivary IgA	PLA	17.94 ± 1.80	18.17 ± 1.79
(μg/mL)	BAE	15.28 ± 1.84	21.69 ± 2.27*

*Results are mean ± SEM, n = 17 individuals per nutritional group*.

* *p < 0.05 represents statistical difference from week 1 values*.

### Interleukin-10 Mediates Antioxidant Properties

Since IL-10 is shown to regulate the antioxidant/anti-inflammatory cellular status (50), we explored the putative antioxidant properties of IL-10 to facilitate exercise-induced oxidative stress/acute inflammation. Utilizing a mouse differentiated myotubule cell-line we examined the ability of IL-10 to modulate cellular oxidative stress evoked by AAPH.

#### AAPH-Induced Cellular ROS Generation

**Figure 6:**
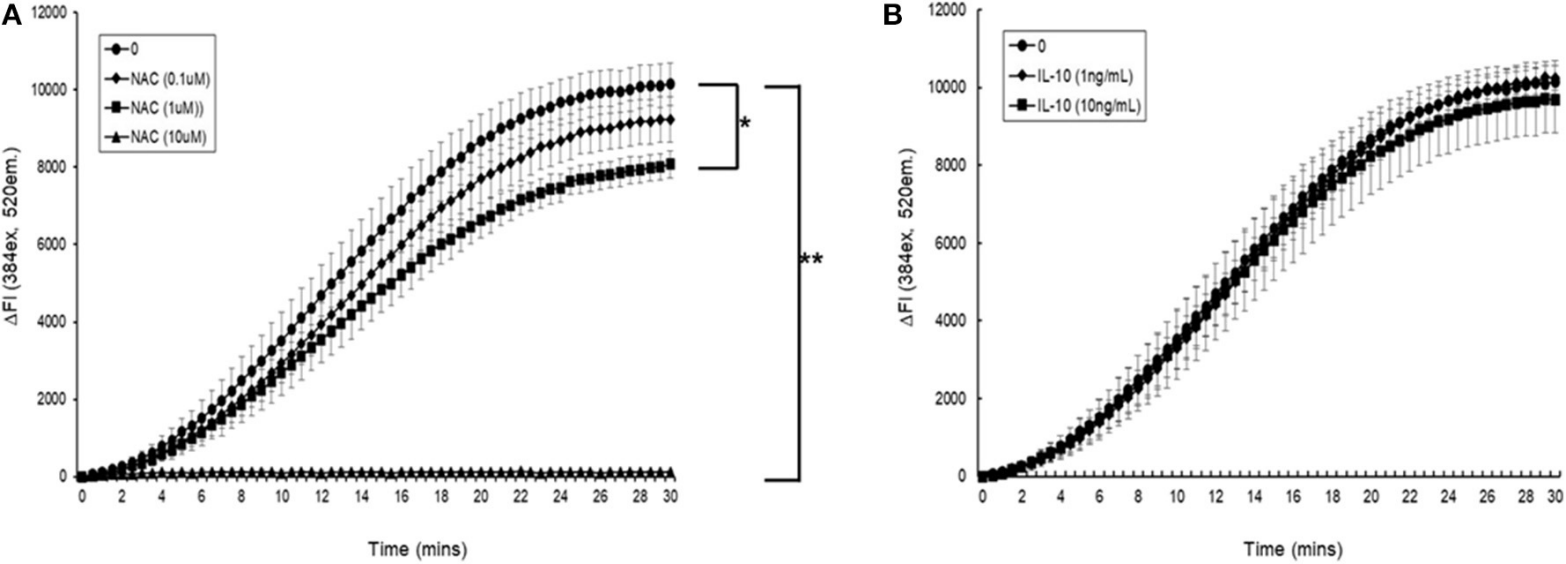
The direct effect of **(A)** N-acetyl cysteine (NAC) or **(B)** IL-10 on AAPH-stimulated reactive oxygen species (ROS) generation in a mouse differentiated myotubule cell-line. Results are presented as a change in fluorescence intensity (ΔFI, excitation [Ex.] 384 nm, emission [Em.] 520 nm) (ΔFI) over 30 min. Results are mean ± SEM of five separate experiments. *p < 0.05 and **p < 0.001 represents statistical difference from AAPH control.

**Figure 7:**
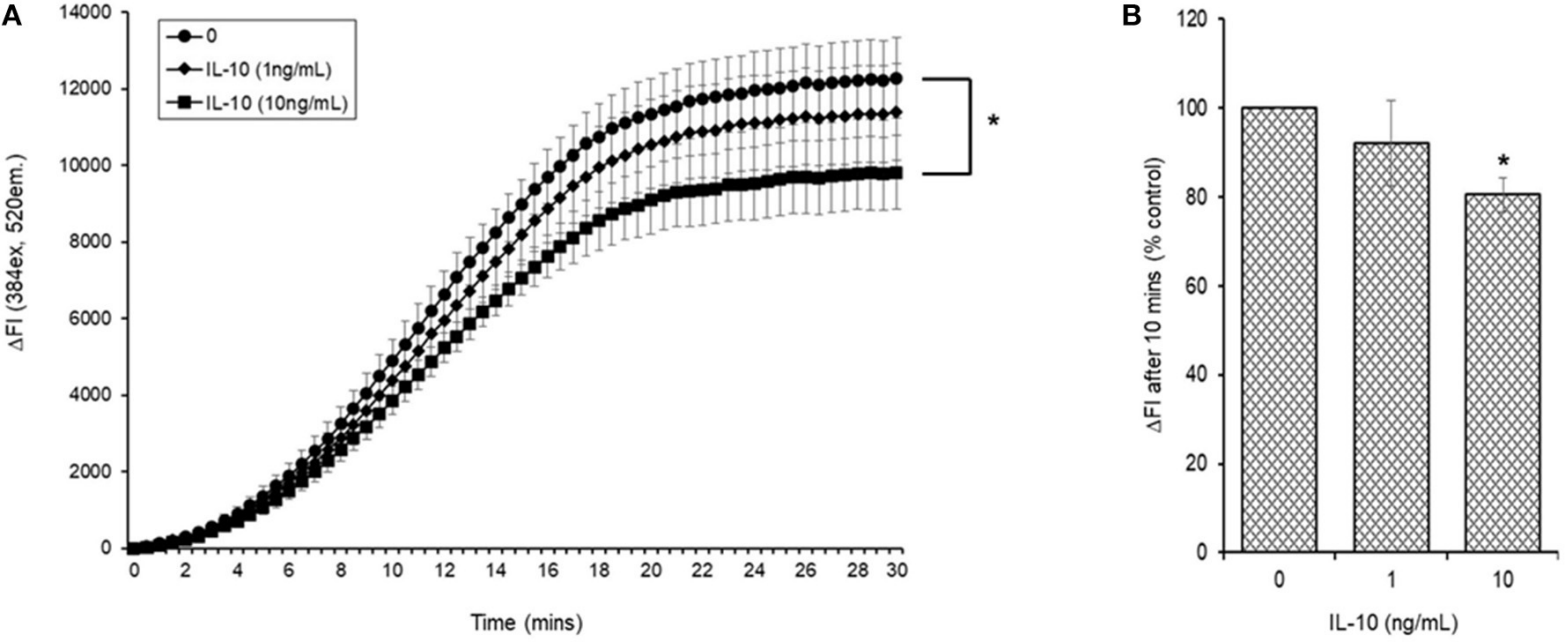
AAPH-stimulated reactive oxygen species (ROS) generation in a differentiated mouse myotubule cell-line after 24 h incubation in the absence or presence of IL-10. Results are presented as a **(A)** change in fluorescence intensity (ΔFI; excitation [Ex.] 384 nm, emission [Em.] 520 nm) over 30 min and **(B)** mean change in fluorescence intensity after 10 min (% AAPH control). Results are mean ± SEM of six separate experiments. *p < 0.05 represents statistical difference from AAPH control.

Initial experiments demonstrated that simultaneous addition of 5 mM AAPH with NAC to carboxy H_2_DCFDA loaded myotubules caused a dose-dependent neutralization of AAPH-induced cellular ROS generation (Figure 6A). Simultaneous addition of IL-10 and 5 mM AAPH demonstrated that IL-10 had no direct scavenging action of the AAPH-induced ROS by myotubules (Figure 6B). In contrast, pre-incubation of myotubules with IL-10 for 24 h prior to evoking ROS caused a dose-dependent inhibition of AAPH-induced ROS generation (Figure 7A). Furthermore, expression of results as ΔFI_10mins_ (Figure 7B) revealed that pre-incubation of myotubules with 10 ng/mL IL-10 for 24 h caused a significant (*P* = 0.007) 20% inhibition of AAPH-induced intracellular ROS generation (4,884.5 ± 564.3 vs. 3,855.6 ± 377.1 ΔFI_10min_, 0 vs. 10 ng/mL IL-10).

#### AAPH-Induced Cellular Protein Carbonyls (Figure 8)

**Figure 8:**
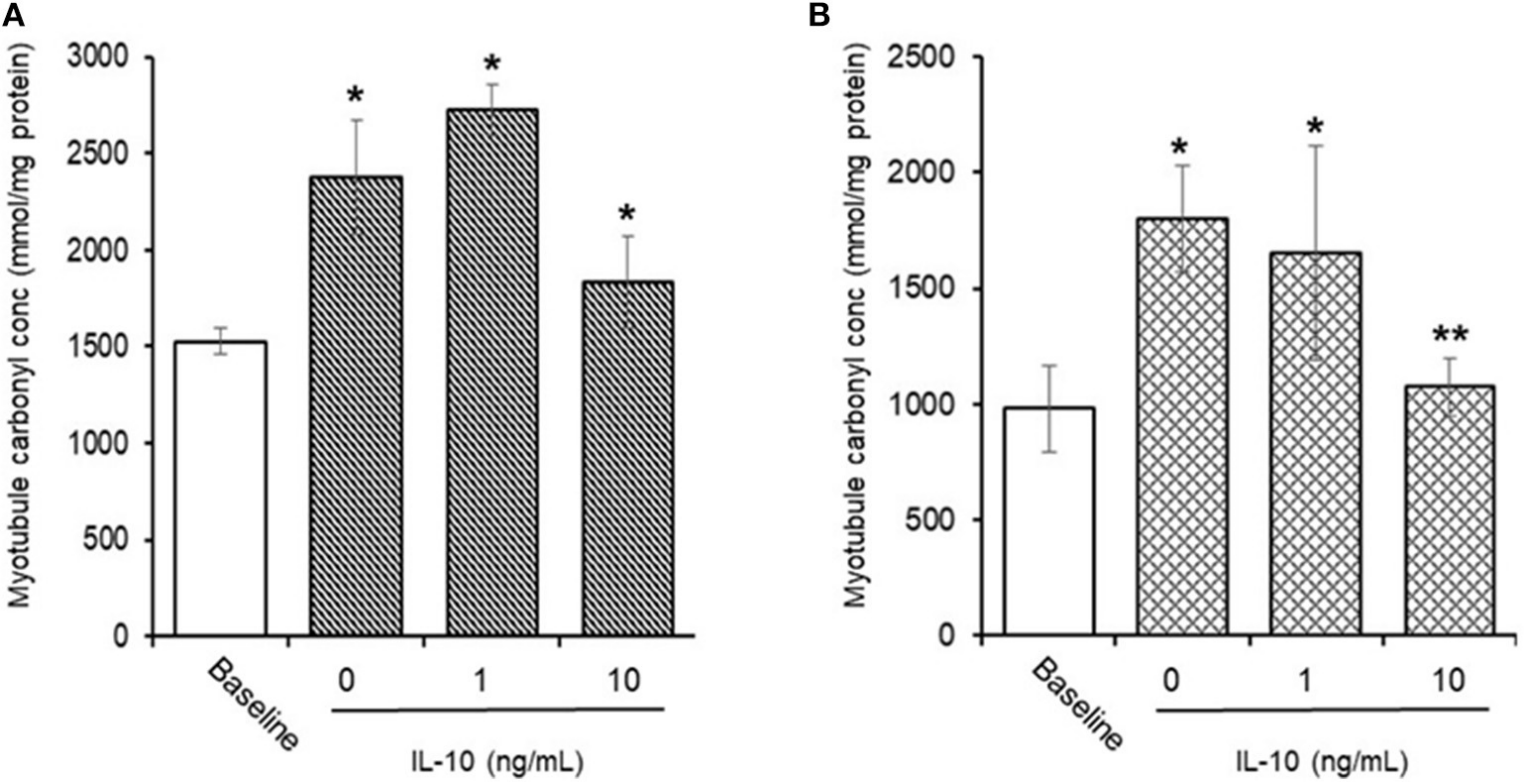
Direct **(A)** or indirect **(B)** antioxidant action of IL-10 on AAPH-stimulated protein carbonyl content in differentiated mouse myotubules. Results are mean ± SEM of six separate experiments. *p < 0.05 and **p < 0.05 represents statistical difference from un-stimulated (baseline) or AAPH-stimulated myotubule protein carbonyl content, respectively.

Preliminary experiments found that 25 mM AAPH caused a significant (*p* < 0.01) increase in total protein carbonyls after 60 min (1,526.3 ± 67.9 vs. 2,381.2 ± 295.3 mmoles/mg protein, baseline vs. AAPH). Simultaneous incubation of myotubules with 25 mM AAPH and IL-10 had no direct effect (Figure 8A), whereas co-incubation of myotubules with 25 mM AAPH plus 10 μM NAC caused a significant (*p* < 0.05) increase in myotubule protein carbonyl levels (1,664.1 ± 244.8 mmoles/mg proteins). In contrast, pre-incubation of myotubules with IL-10 for 24 h before evoking an oxidative stress, caused a dose-dependent inhibition of the AAPH-induced increase in protein carbonyl levels (Figure 8B). Pre-incubation of myotubules with 10 ng/mL IL-10 mediated a 33% (1,073.2 ± 126.8 mmoles/mg protein, *P* < 0.05) decrease in AAPH-induced myotubule carbonyl concentration (1,799.2 ± 226.4 mmoles/mg protein).

#### AAPH-Induced Cellular MDA (Figure 9)

Incubation of myotubules with 25 mM AAPH for 60 min caused a significant (*P* < 0.05) increase in total cellular MDA levels (0.05 ± 0.02 vs. 0.80 ± 0.16 nM, baseline vs. AAPH). Co-incubation of myotubules with 25 mM AAPH and IL-10 had no direct effect on the AAPH-induced increase in myotubule MDA levels: 0.88 ± 0.22 vs. 1.18 ± 0.42 nM, AAPH control vs. AAPH plus 10 ng/mL IL-10 (Figure 8A), whereas co-incubation of myotubules with 25 mM AAPH and 10 μM NAC resulted in a significant 59% reduction (*P* < 0.05) in myotubule MDA concentrations (0.37 ± 0.03 nM). Pre-incubation of myotubules with IL-10 for 24 h before evoking an oxidative stress with 25 mM AAPH resulted in a dose-dependent inhibition of the AAPH-induced increase in cellular MDA concentration (Figure 8B). Pre-incubation of myotubules with 10 ng/mL IL-10 caused a significant 40% (0.22 ± 0.01 nM. *P* < 0.01) decrease, respectively in 25 mM AAPH-induced myotubule MDA concentration (0.37 ± 0.03 nM).

**Figure 9:**
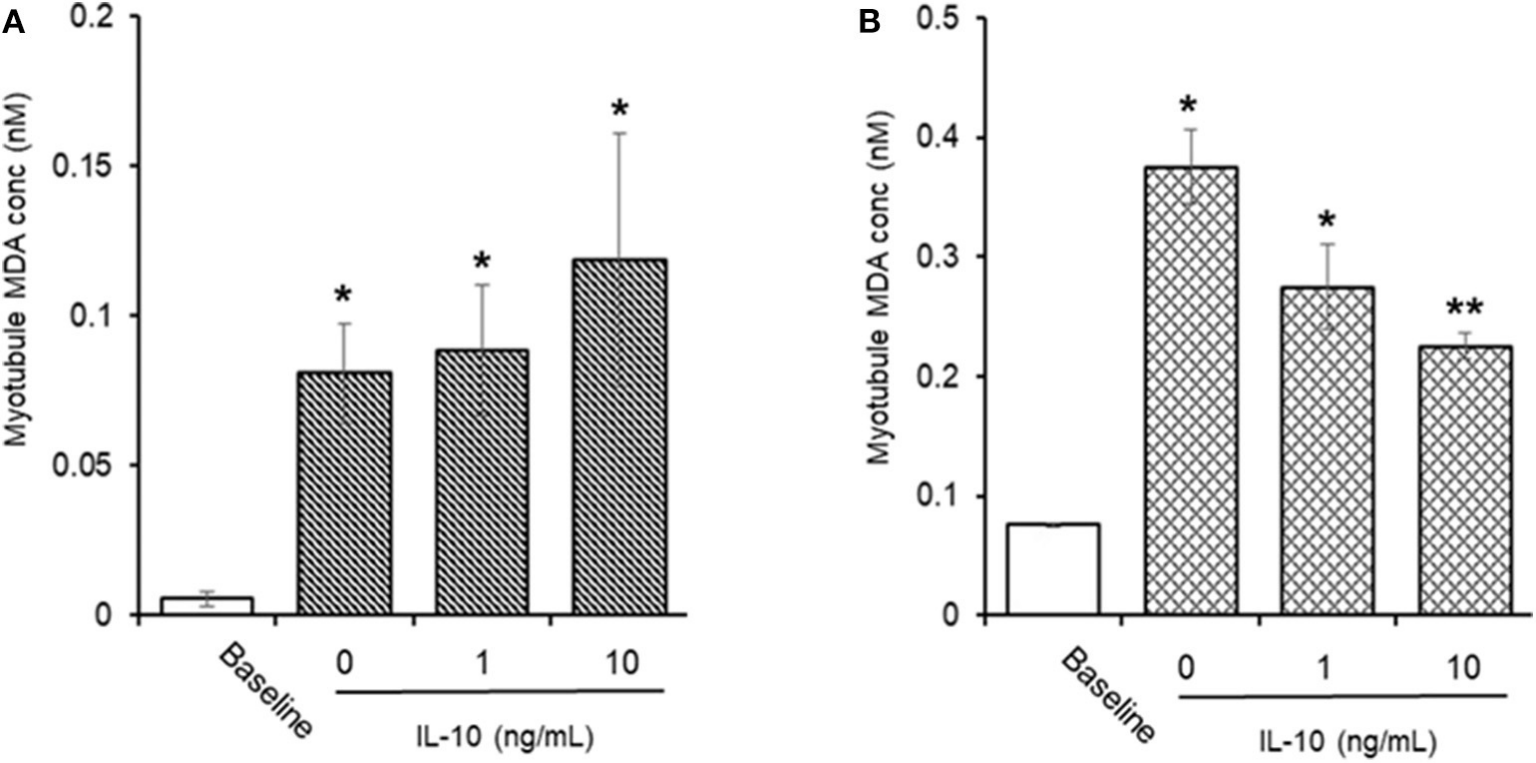
Direct **(A)** or indirect **(B)** antioxidant effect of IL-10 on AAPH-stimulated malondialdehyde (MDA) content in differentiated mouse myotubules. Results are mean ± SEM of 6 separate experiments. *p < 0.05 and **p < 0.05 represents statistical difference from un-stimulated (baseline) or AAPH-stimulated myotubule MDA content, respectively.

## Discussion

Consumption of anthocyanin-rich supplements for a set period of time before exercise has been shown to be an effective way of alleviating the detrimental aspects of exercise, such as oxidative stress, whilst maximizing exercise-induced physiological health benefits and ergonomic adaptive benefits (33, 51). A previous study by us (41) shows that the consumption of blackcurrant anthocyanin-rich extract containing 3.2 mg/kg total anthocyanins just 1 h before a 30-min rowing exercise (70% VO_2max_) facilitated the recovery from exercise-induced oxidative stress. Here we found the daily consumption of the BAE for 5 weeks contributed to the maintenance (or even enhancement) of cellular antioxidant/anti-inflammatory properties that support exercise recovery. The notion that the consumption of anthocyanins before exercise supports cellular antioxidant/anti-inflammatory properties to facilitate exercise recovery has been reported in other timed nutrition-exercise studies. For example, athough using different exercise methods from us, the timed consumption of either anthocyanin-rich “Montmorency” cherry (52) or chokeberry (53) juices during exercise training programmes improved the recovery from exercise-induced oxidative stress and inflammation compared with consumption of matched placebos. The authors attributed this to an anthocyanin-driven increase in antioxidant capacity. In contrast, however, daily consumption of an anthocyanin-rich extract containing the equivalent of 100 mg anthocyanins during a 6 week set aerobic training programme showed no overall change in exercise recovery compared with consumption of a placebo (54). Whilst the underlying mechanism is unknown, i.e., the efficacy may be due to downstream anthocyanin metabolites or an accumulation of anthocyanins and/or derived metabolites within tissues (6), the inclusion of anthocyanin-rich foods/drinks/supplements (e.g., blackcurrant anthocyanins) as part of a competitive or non-competitive exercise training regime has shown potential benefits. It is becoming apparent that the nutritional composition/timing, the exercise type/intensity/duration, the relative fitness of the individual, and the resulting anthocyanin-induced cell bioactivity are all important considerations in designing a nutritional strategy to complement and maximize the desired exercise-induced health and ergonomic outcomes.

Intervention studies report that the long-term consumption of certain polyphenol-rich foods/supplements, including anthocyanins, results in the up-regulation of innate immunity that is linked to a reduced risk of developing age-related diseases such as obesity and cardiovascular disease (11–13). In this study, we found that the daily consumption of the blackcurrant extract containing 3.2 mg/kg total anthocyanins for 5 weeks enhanced the expression of immune factors associated with the maintenance of innate immune defenses (e.g., salivary BD2 and IgA) and enhanced cellular anti-inflammatory/antioxidant properties (e.g., IL-10). Since exercise-induced oxidative stress has been shown to cause fluctuations in mucosal immunity (55, 56), it is feasible that an anthocyanin-mediated up-regulation of the secretory immune components (such as BD2 and IgA, observed over this 5 week period), may preserve this first line of innate defense to reduce the risk of opportunistic infection (57, 58). Indeed, a previous study by Baralic et al. (59) showed that astaxanthin supplementation for 90 days in soccer players before an exercise trial resulted in an increased salivary IgA. IgA is identified as having a protective role in counteracting exercise-induced impairment of mucosal defenses. It is also feasible that an up-regulation of salivary BD2 may have the same defensive outcome, since in addition to its antimicrobial property it has been implicated in the regulation of inflammation in the maintenance of bacterial homeostasis in the oral cavity (60).

IL-10 is an immune modulator that demonstrates both anti-inflammatory and antioxidant properties (50). Specifically, perturbations in the cellular redox- and oxidant-mediated signaling pathways (e.g., in response to physical activity/exercise) have been shown to produce a dynamic microenvironment that is critical for the anti-inflammatory/antioxidant action of IL-10. We found that the daily consumption of BAE for 5 weeks caused a significant increased plasma IL-10. Since IL-10 is associated with supporting the cell's antioxidant/anti-inflammatory properties, it could be speculated that this increase in IL-10 may have contributed to the enhanced resolution of exercise-induced acute inflammatory response, and to the reduction in plasma ROS-generating capability observed after 2 h of recovery at week 6. The putative role of anthocyanins in up-regulating innate immunity is supported by other nutrition studies. McAulty et al. (61) found that the daily consumption of blueberries for 6 weeks elevated innate immune indices, including plasma IL-10. Furthermore, consumption of foods rich in anthocyanins has also been shown to increase the IL-10: TNFα ratio, promoting a shift toward an anti-inflammatory/antioxidant status (62–65). Nutritional intervention studies targeting the amelioration of chronic inflammation found that administration of other polyphenolic compounds suppressed inflammation by creating an anti-inflammatory/antioxidant environment. For example Carito et al. (66) found that 10 days of daily intraperitoneal administration of an olive polyphenolic extract in a mouse model of paw inflammation partially restored the expression of IL-10, with a concomitant shift to an anti-inflammatory status. Furthermore, consumption of berry fruit foods/supplements rich in anthocyanins, such as blueberry, have been shown to alleviate symptoms of trinitrobenzene sulfonic acid (TNBS)-induced colitis (67) and cyclophosphamide-induced cardiac left ventricle inflammation (62) by increasing the anti: pro (IL-10: TNFα) inflammatory ratio. Similarly, a study of three-times-daily consumption of a berry maqui extract rich in the anthocyanin delphinidin for 4 weeks in healthy overweight individuals who smoked showed an overall reduction in biomarkers of oxidative stress (68). These findings support that the long-term consumption of foods rich in anthocyanins may serve to create an anti-inflammatory/antioxidant microenvironment, which controls underlying chronic inflammation and oxidative stress, and promotes tissue repair processes. In our current study, we have revealed that the daily consumption of BAE for 5 weeks significantly improved the time-dependent recovery from the 30 min moderate exercise. Although we detect no significant change in overall plasma antioxidant capacity (using FRAP assay), a positive correlation with plasma IL-10 was observed on week 6. These data suggest that the observed increase in plasma IL-10 may have played a role in the enhanced reduction of post-exercise plasma oxidative-generating capacity observed in week 6, potentially through its reported additional antioxidant ability.

Regular exercise (aerobic or resistance) has been shown to increase plasma IL-10 concentrations (69, 70). Since IL-10 acts as a redox modulator, the increase in exercise-induced IL-10 may not only be the consequence of repeated exercise-induced shifts in cellular redox status. It may also play an important hormetic role in the up-regulation of adaptive cellular systems (50, 71, 72). In our study, the efficacy of IL-10 in a myotubule cell model showed that it had no direct antioxidant action on AAPH-induced oxidative stress. However, a 24 h incubation of the myotubules in the presence of IL-10 revealed a dose-dependent attenuation of AAPH-induced oxidative stress. These results support a role of IL-10 in alleviating exercise-induced oxidative stress in skeletal muscle (73–75), and they support further that the increase in plasma IL-10 observed after the daily consumption of the BAE for 5 weeks may contribute to alleviating skeletal muscle oxidative stress induced by the 30 min row exercise. In similar cell studies, IL-10 (71) reported antioxidant properties in regulating intestinal epithelial cell redox equilibrium. They found that a 24 h incubation of the CaCo2 intestinal epithelial cell-line in the presence or absence of IL-10 reversed the oxidative damage in lipids and proteins. Similarly, IL-10 antioxidant properties have been demonstrated in monocytes (76), cardiomyocytes (77), and leukocyte/endothelial interactions (63). These findings suggest that the antioxidant properties of IL-10 may be evident in tissues that have been exposed to oxidative/pro-inflammatory conditions, but through mechanisms that do not appear to directly alter the antioxidant enzyme activities. The increase in plasma IL-10 observed in our study was not due to the exercise itself, because the study participants exhibited similar exercise patterns and intensity which did not change over the 5 week study period. In addition, only the participants who consumed BAE daily for 5 weeks displayed an increase in plasma IL-10 over the 5 weeks. We therefore conclude that the blackcurrant-derived anthocyanins contained in the blackcurrant extract may have been responsible for the observed increase in plasma IL-10 (and possibly other yet undiscovered cellular redox regulators), which contributed to a shift in the antioxidant/anti-inflammatory cell microenvironment, and supported exercise recovery and the maintenance of innate immune defenses.

There is growing evidence that consumption of polyphenolic-rich foods/supplements may alleviate the detrimental effects of exercise (e.g., fatigue, eccentric muscle damage, delayed recovery) and facilitate adaptive ergonomic and health benefits (33, 51). However, the underlying cellular mechanisms appear variable, and involve an interplay between antioxidant and inflammatory signaling systems. Furthermore, it is currently unknown how long-term consumption of polyphenolic and/or anthocyanin-rich foods results in the maintenance of an effective cellular anti-inflammatory/antioxidant microenvironment, and especially how this may involve the up-regulation of mucosal immunity (BD2, IgA) and cellular redox modulators, such as IL-10. Although still unknown and hence of interest, a potential underlying mechanism could include the ability of anthocyanins, through their putative chemical electrophilic properties (78), to mediate changes in cell redox perturbations that result in the up-regulation of adaptive anti-inflammatory responses [e.g., involving IL-10, (50)] and, as we observed in this study with an improved resolution of TNFα and enhanced IL-10 levels following 5 weeks of BAE consumption. This notion is supported by a number of cell studies (23, 24, 79) that show that anthocyanin-induced increases in cellular antioxidant enzyme systems involve redox-initiated transcription of nrf2/ARE. It is also feasible there is a relationship between oxidative stress-driven nrf2/ARE transcription and the expression of anti-inflammatory mediators, such as TNFα and IL-10 (80), which regulates cell redox homeostasis to favor an anti-inflammatory/antioxidant cellular microenvironment. It was not our aim in this study to evaluate whether adaptive anti-inflammatory or antioxidant responses underlie the mechanism of action of long-term consumption of BAE and hence we did not examine transcription pathway activation, nor cell antioxidant enzyme expression or activity. However, other cell studies using anthocyanins (including blackcurrant anthocyanins) have demonstrated that they can act as electrophiles (78) within cells to activate the nrf2/ARE-driven transcription of several antioxidant enzymes (9). Therefore, it is feasible that the daily consumption of the BAE for 5 weeks could result, with exercise, in the activation and regulation of adaptive cellular pathways (possibly involving a shift in the cells' redox status), which contributes to a change in the anti-inflammatory/antioxidant cell microenvironment that further limits cell stress/damage and maintains and facilitates cell and tissue function.

In conclusion, our findings support that the daily consumption of BAE (containing 3.2 mg/kg) for 5 weeks serves to maintain and/or enhance the exercise recovery effectiveness of an acute single consumption of BAE by promoting a beneficial/protective antioxidant/anti-inflammatory cellular environment that facilitates exercise recovery. Our data add to the accumulating knowledge of how polyphenolic compounds support health-promoting cellular processes. Understanding the interplay between nutritional timing of macronutrients such as plant polyphenolic compounds (e.g., anthocyanins) and exercise to maximize beneficial adaptive cellular events may ultimately enable the design and implementation of nutrition-exercise training strategies that specifically target the desired adaptive ergonomic, performance and human health outcomes.

## Data Availability Statement

The datasets generated for this study are available on request to the corresponding author.

## Ethics Statement

The studies involving human participants were reviewed and approved by HDEC New Zealand. The patients/participants provided their written informed consent to participate in this study.

## Author Contributions

RH and SH secured the MBIE funding to support this study, interpreted results, and approved the final version of the manuscript submitted. RH, SH, and KL designed the study. SH gained human ethical approval from the Northern Ethical Regional Committee, Hamilton, New Zealand. KL recruited the participants and was the trial coordinator for the human trials and revised the manuscript critically for important intellectual content. SH, KL, RW, GS, and DL carried out the experimental analysis on collected blood samples. SH, NN, and RH drafted the manuscript. Neither MBIE nor the New Zealand Blackcurrant industry had any role in the study concept, design, data collection, data analysis, decision to publish, or preparation of the manuscript.

### Conflict of Interest

The authors are employees of the New Zealand Institute for Plant and Food Research Ltd, which has no royalty agreement associated with sales of New Zealand blackcurrant products. The authors declare that there is no individual personal financial relationship.
